# Torsion of a Pedunculated Subserosal Leiomyoma: A Rare Cause of Acute Abdominal Pain

**DOI:** 10.7759/cureus.48414

**Published:** 2023-11-06

**Authors:** Manjusha Agrawal, Ketki S Dantkale, Deepika Dewani, Pravin Karwade, Nidhi Goyal

**Affiliations:** 1 Obstetrics and Gynecology, Jawaharlal Nehru Medical College, Datta Meghe Institute of Higher Education and Research (Deemed to be University), Wardha, IND

**Keywords:** pedunculated subserosal leiomyoma, color doppler, torsion, myomectomy, acute abdomen

## Abstract

Uterine leiomyomas are benign uterine tumors arising from the smooth muscle cells of the myometrium. Most of them are asymptomatic, and rarely do they present with symptoms like infertility, abdominal distension, and acute abdomen. According to experts, the most common cause of acute abdomen is torsion of a pedunculated subserosal leiomyoma, which is an extremely rare and life-threatening surgical emergency. Here, we discuss a case of torsion of a subserosal leiomyoma where the patient, a 40-year-old female with severe abdominal pain, was misdiagnosed with a twisted ovarian cyst. Ultrasonography and contrast-enhanced computed tomography (CECT) revealed a right-sided ovarian tumor and a twisted subserosal myoma, respectively. Thus, surgical intervention with myomectomy was done.

## Introduction

Uterine leiomyomas are benign gynecological tumors of the female genital tract frequently seen in childbearing age women with an incidence of about 20-40% [1]. Symptoms include menorrhagia, abnormal vaginal bleeding, dysmenorrhea, pelvic pain, and bowel and bladder pressure symptoms. Acute-onset severe abdominal pain is usually related to complications like red degeneration, infection, the process of expulsion of submucous pedunculated myoma, or torsion of a pedunculated subserosal myoma [2]. However, torsion of a pedunculated uterine leiomyoma is extremely rare, with a reported incidence of less than 0.25% [3]. Torsion of a pedunculated leiomyoma is considered an emergency due to the risk of ischemic gangrene and reactive peritonitis [4]. Since it is difficult to diagnose a twisted pedunculated leiomyoma solely on the basis of clinical findings, the judicious use of radiological imaging becomes a fundamental tool for achieving a precise diagnosis [5]. The first line of radiological imaging technique in such cases is ultrasonography. However, due to its low specificity, other imaging modalities, such as computed tomography (CT) scan or magnetic resonance imaging (MRI), are recommended for accurate diagnosis. This case reports a reproductive age woman who presented with acute abdomen and required immediate surgical intervention for a twisted pedunculated subserosal leiomyoma.

## Case presentation

A 40-year-old P2L2A1 with two previous vaginal deliveries presented to casualty in emergency hours with complaints of sudden-onset severe abdominal pain precisely on the right side for two days. The pain was colicky type, was intermittent, and increased on movement and defecation. It radiated to the lower back and was mildly relieved by using oral analgesics and on rest. The pain was accompanied by nausea and two episodes of vomiting. She had no history of dysuria, urgency, bleeding per vaginum, or any discharge. Her last menstrual period was three weeks ago and was regular with average flow.

Upon examination, she was febrile (temperature: 100.6 degrees Fahrenheit) and had tachycardia (110 beats/minute). Her blood pressure was 110/74 mmHg. Her cardiopulmonary examination did not reveal any abnormality. She had tenderness in the lower abdomen, which was more pronounced in the right iliac fossa. A suprapubic firm to cystic mass corresponding to the size of that of a 14-week gravid uterus, more deviated towards the right side, was felt per abdominally. Guarding and rigidity were not present. Bowel sounds were normal. On bimanual examination, a firm to cystic, tender mass of size 11 x 10 cm was felt through the anterior and right lateral fornix. The uterus could not be felt separately. Bilateral fornices were tender.

Laboratory tests showed an elevated total leukocyte count of 15400/cumm with normal hemoglobin (11.6 gm%) (Table 1). The urine pregnancy test was found to be negative. Other biochemical evaluations and urine analyses showed no abnormalities. A clinical provisional diagnosis of a twisted ovarian cyst was made.

**Table 1:** Blood investigation of the patient CBC: complete blood count; LFT: liver function test; HB: hemoglobin; TLC: total leukocyte count; PLT: platelet count; SGOT: serum glutamic oxaloacetic transaminase; SGPT: serum glutamic pyruvic transaminase; KFT: kidney function test: RBS: random blood glucose; mg/dl: milligram per liter; mmol/l: millimole per liter

Sr. No.	Investigation		Measured value	Reference value
1	CBC	HB	11.6 gm%	12-15 gm%
		TLC	15400/cumm	4500-11000/cumm
		PLT	2 L/cumm	150,000-450,000 L/cumm
2	LFT	Alkaline phosphatase	124 U/L	44-147 U/L
		SGOT	30 IU/L	8-45 IU/L
		SGPT	42 IU/L	7-56 IU/L
		Total protein	4.9 g/dl	6-8.3 g/dl
		Globulin	2.6 g/dl	2-35 g/dl
		Albumin	3.2 g/dl	3.4-5.4 g/dl
		Total bilirubin	0.5 mg/dl	0.8-1 mg/dl
3	KFT	Urea	15 mg/dl	5-20 mg/dl
		Creatinine	0.7 mg/dl	0.6-1.1 mg/dl
		Sodium	137 mmol/l	136-145 mmol/l
		Potassium	4.6 mmol/l	3.6-5.2 mmol/l
4	RBS		90 mg/dl	Less than 140 mg/dl

**Figure 1:**
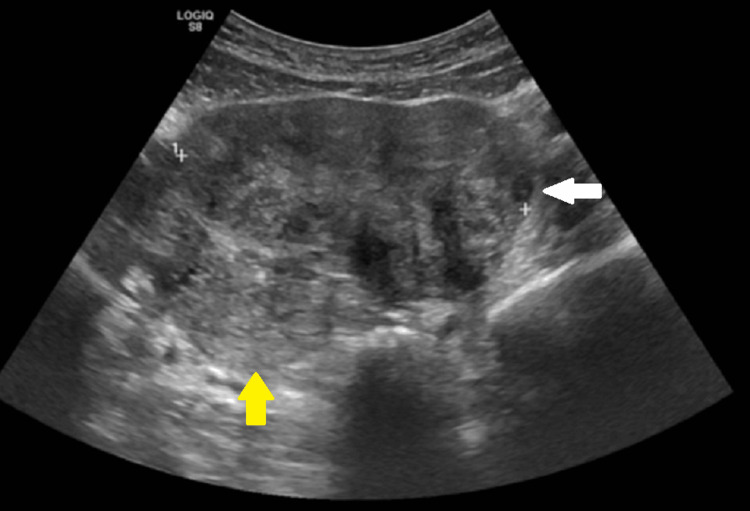
A large 11 x 10 cm mass (white arrowhead) with heterogeneous echotexture seen closely adjacent to the uterine fundus (yellow arrowheads) on ultrasonography

Ultrasonography revealed an adnexal mass on the right side of size 11 x 10 cm with heterogeneous echogenicity. The right ovary could not be separately differentiated from the adnexal mass. The left ovary was normal. On color Doppler, the twisted pedicle was visible with decreased arterial and venous blood flow in the parauterine mass (Figure 1). 

**Figure 2:**
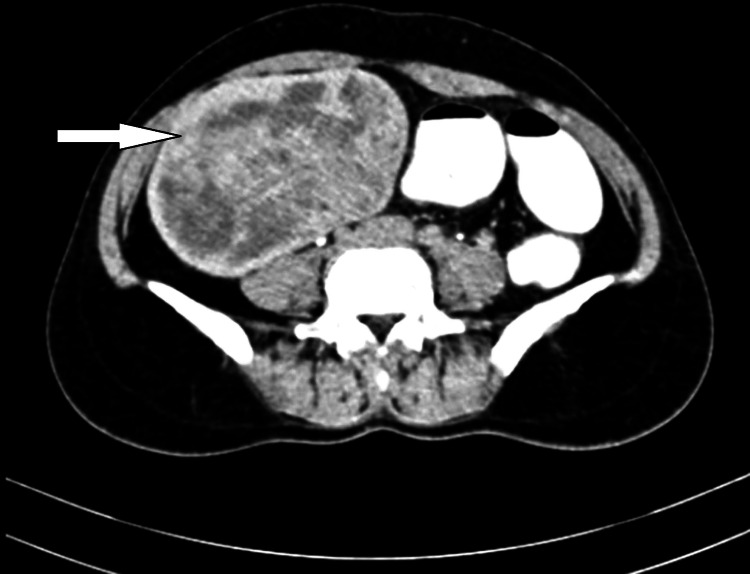
A large 12 x 10 cm heterogeneous mass (white arrow) with necrotic areas seen in the subserosal myoma

**Figure 3:**
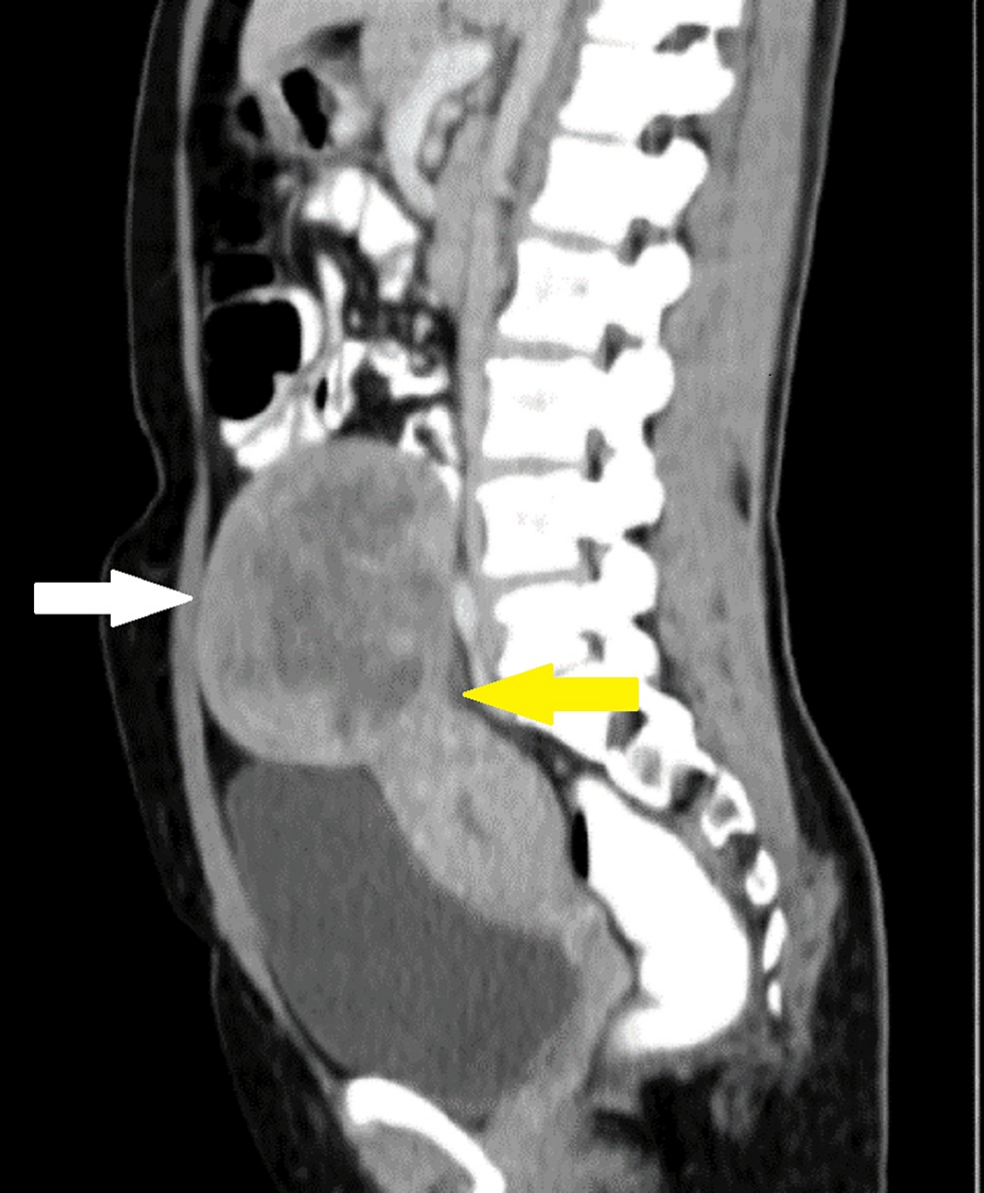
Sagittal section of a CT scan of a leiomyoma (white arrow) of size 12 x 10 x 7 cm with a stalk-like connection (yellow arrow) between the uterus and mass CT: computed tomography

On contrast-enhanced computed tomography (CECT) of the abdomen and pelvis, a 12 x 10 x 7 cm heterogeneous mass in the right adnexa extending from the right iliac to the right lumbar region was seen. It was causing a mass effect in the form of lateral displacement of bowel loops (Figure 2 and Figure 3).

**Figure 4:**
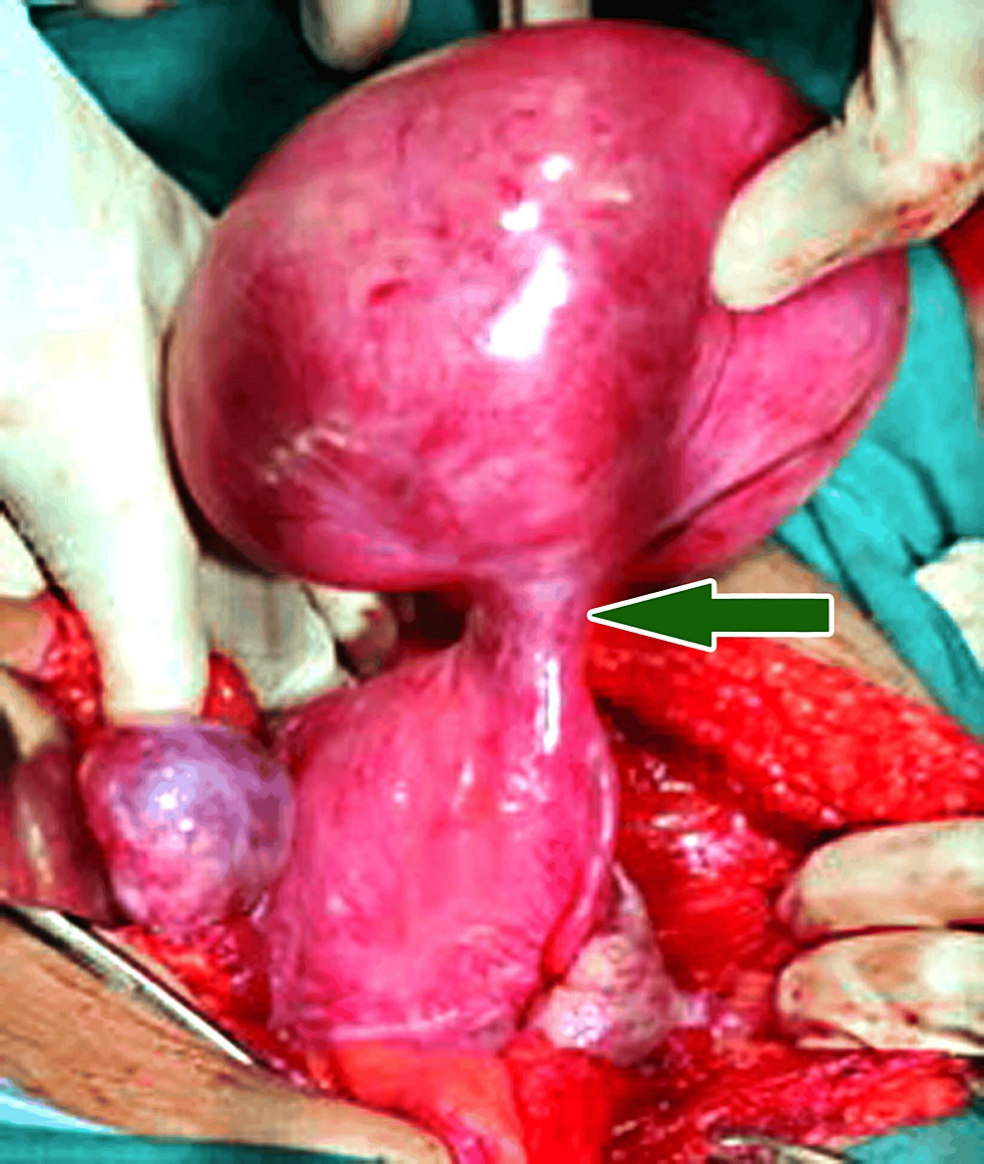
Torsion of a pedunculated subserosal leiomyoma by 270 degrees (green arrow)

The patient was immediately shifted for emergency laparotomy. Intraoperative examination confirmed the diagnosis of torsion of a subserosal leiomyoma which was located on the fundus anteriorly towards the right cornu of size 12 x 9 x 8 cm. It was twisted by 270 degrees over a 2-3 cm pedicle (Figure 4) and had minimal gangrenous changes.

**Figure 5:**
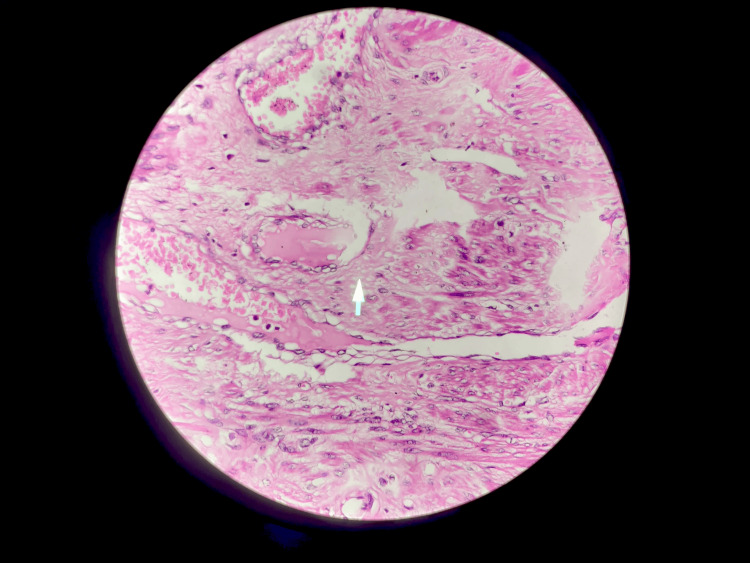
Histopathological view of leiomyoma with a necrotic focus

Detorsion of the leiomyoma and myomectomy were done, and the specimen was sent for histopathological examination. Both ovaries were normal. Endometrial curettage was done to rule out any endometrial pathology. Histopathology confirmed a benign necrotic leiomyoma with no signs of malignancy, and the endometrial biopsy showed endometrial cells in the secretory phase. Figure 5 represents the histopathological view of leiomyoma with a necrotic focus.

## Discussion

In symptomatic patients with normal ovary on ultrasound, or when associated with pregnancy, confirmation or exclusion of torsion of an adnexal mass is of paramount importance [6]. Hence, it is essential to take a multidisciplinary approach in a patient presenting with an acute condition for prompt diagnosis and management.

Pedunculated subserosal uterine leiomyomas can be extremely challenging to diagnose clinically. In most cases, patients are asymptomatic. Abnormal uterine bleeding and pelvic pain are the most typical symptoms of leiomyomas [7]. Acute pain in the abdomen as a symptom in leiomyoma patients is uncommon. Infection, red degeneration, the process of expulsion of submucous pedunculated myoma protruding through the external os, pressure caused by the leiomyoma between the uterus and sacrum, and torsion of a pedunculated subserosal uterine leiomyoma are complications of leiomyomas that are linked to acute abdominal pain [8].

Since torsion of a pedunculated subserosal leiomyoma lacks truly distinct clinical signs and symptoms, its accurate diagnosis is challenging to make prior to surgery. However, imaging modalities may aid its identification, and these techniques appear to be more important than a clinical examination. A large solid mass that is situated in proximity to the uterus and ovaries might be seen on a CT scan. MRI can also occasionally reveal the pedicle that joins the uterus to the mass that seems to be a necrotic leiomyoma [9]. Moreover, uterine masses with twisted peduncles can be seen on abdominal and Doppler ultrasonography by assessing the blood flow which can help with the preoperative diagnosis of twisted pedunculated subserosal uterine leiomyoma [10].

Surgery is also ideal to treat twisted pedunculated subserosal uterine leiomyoma. Myomectomy, which can be carried out by laparoscopy or laparotomy, is the preferred therapy option for pedunculated subserosal leiomyomas in women of reproductive age [11].

## Conclusions

Torsion is a rare complication of pedunculated subserosal uterine leiomyoma. It is extremely difficult to diagnose it prior to surgery, even with the availability of diagnostic modalities. Therefore, in patients presenting with acute abdomen who have uterine leiomyoma and adnexal masses, torsion should be evaluated as one of the differential diagnoses. A laparotomy or laparoscopic approach instead of conservative management is always preferred for such patients to avoid further consequences.
